# Identifying Free-Living Physical Activities Using Lab-Based Models with Wearable Accelerometers

**DOI:** 10.3390/s18113893

**Published:** 2018-11-12

**Authors:** Arindam Dutta, Owen Ma, Meynard Toledo, Alberto Florez Pregonero, Barbara E. Ainsworth, Matthew P. Buman, Daniel W. Bliss

**Affiliations:** 1 School of Electrical, Computer and Energy Engineering, Arizona State University, Tempe, AZ 85281, USA; owenma@asu.edu (O.M.); d.w.bliss@asu.edu (D.W.B.); 2 College of Health Solutions, Arizona State University, Phoenix, AZ 85281, USA; mltoledo@asu.edu (M.T.); bainswor@asu.edu (B.E.A.); mbuman@asu.edu (M.P.B.); 3 Departamento de Formación, Pontificia Universidad Javeriana, Bogotá D.C. 110231, Colombia; floreza@javeriana.edu.co

**Keywords:** physical activity classification, free-living, GENEactiv accelerometer, machine learning, Gaussian mixture model, hidden Markov model, wavelets

## Abstract

The purpose of this study was to classify, and model various physical activities performed by a diverse group of participants in a supervised lab-based protocol and utilize the model to identify physical activity in a free-living setting. Wrist-worn accelerometer data were collected from (N=152) adult participants; age 18–64 years, and processed the data to identify and model unique physical activities performed by the participants in controlled settings. The Gaussian mixture model (GMM) and the hidden Markov model (HMM) algorithms were used to model the physical activities with time and frequency-based accelerometer features. An overall model accuracy of 92.7% and 94.7% were achieved to classify 24 physical activities using GMM and HMM, respectively. The most accurate model was then used to identify physical activities performed by 20 participants, each recorded for two free-living sessions of approximately six hours each. The free-living activity intensities were estimated with 80% accuracy and showed the dominance of stationary and light intensity activities in 36 out of 40 recorded sessions. This work proposes a novel activity recognition process to identify unsupervised free-living activities using lab-based classification models. In summary, this study contributes to the use of wearable sensors to identify physical activities and estimate energy expenditure in free-living settings.

## 1. Introduction

Engaging in sufficient amounts of physical activity (PA) is associated with decreased risk of premature mortality from cardiovascular diseases [1,2,3]. The 2008 physical activity guidelines recommend engaging in at least 150 minutes per week of moderate-vigorous physical activity [4]. Without an accurate PA measurement tool, our ability to determine the relationship between physical activity and health, develop effective interventions to promote these healthy behaviors, and evaluate the effectiveness of these interventions, is severely limited. Human beings perform a wide range of complex activities, varying based on age, profession, time of the day and other demographics. Physical activities of many forms including daily household activities, walking, aerobics, and strength training are performed at various intensities (i.e., light, moderate or vigorous), based on the individual. Hence, we need measurement tools to quantify complex human activities accurately, and make necessary interventions to maintain healthy behaviors.

With the advent of wearable and remote sensors, it has become easier to monitor PA due to their objectivity, minimal participant burden and rich data that can be collected for a long period. Human activity recognition using video processing has become a widely studied area of research. Video analysis approaches based on template-based methods [5,6], generative models [7,8,9], and discriminative models [10] have been used to classify complex human activities and gait patterns. However, in this paper we focused on wearable accelerometers, which have become inexpensive, small and lightweight, can gather high-frequency data and can be used by the population across all demographics. Accelerometer-based systems have been used to gain insights about physical activity of all age groups, adolescents [11,12], young adults [13,14,15,16], old adults [17,18,19,20,21,22] and seniors [17,23,24,25]. There are two major research foci in PA monitoring studies: (a) energy expenditure (EE) estimation and (b) activity classification. The traditional approach to EE estimation using accelerometer data is to estimate the intensity (MET value) of an activity through simple linear regression modeling [26]. Another approach attempts to identify the type of activity performed, and calculate EE using knowledge of the activity’s intensity. Current methodological development, especially in signal processing and machine learning techniques, have led researchers to implement alternative frameworks for estimating EE. Of which, methods such as artificial neural network [27], novel estimation framework based on statistical estimation theory [28] and piecewise linear regression model [29], deserve special mention due to their high prediction accuracy. Activity classification studies have been performed with both supervised laboratory and free-living protocols. Most PA classification approaches involve extracting features from raw or processed accelerometer data and using them to identify unique physical activities using machine learning or deep learning based classifiers. Various studies have investigated different types of features and classifiers to identify a wide range of PA, reporting efficiency ranging between 68 and 99% [11,18,19,22,30,31,32,33,34]. All these studies have proposed methods to identify various PA performed by study participants (N≤130) using single [11,18,22,32] or multiple accelerometer units [30,31,35], but strictly in supervised, controlled settings and lack free-living applications. The studies [11,30] that have analyzed a large database of PA (≥20) lack in diversity and total number of study participants (N≤53). Simultaneously, studies [18,32] that dealt with a large number of study participants (N≥100) analyzed less PA (≤12). In this study, however, we have used a larger dataset of (N=152) participants to model 24 PA in a lab-based study using just a single wrist-worn accelerometer. Previous work has generally relied on lab-based activity trials to train and test classification models. However, validity of these previously studied methodologies applied toward free-living contexts is starting to emerge. One such study [36] cross-validated four PA classification models (N=21) and classified four activities in free-living setting from (N=16) participants wearing a wrist-worn accelerometer. Supervised classification was performed with reference to recorded labels using another thigh worn accelerometer. In this study, we use a novel unsupervised framework to identify PA performed by 20 participants in a free -living setting. In the first part of this paper, we train and test classifiers to model physical activities using accelerometer data from lab-based settings. In the second part of the paper, we use the lab-based classification model to identify free -living activities in an unsupervised framework.

The data used in this analysis were gathered through two separate studies conducted in a southwestern university in the USA. The first study provided accelerometer data on structured, lab-based activities that were used to train and validate the proposed machine learning method. The data from the free-living protocol were used to evaluate the developed algorithm in its capacity to estimate activity intensities in a free -living setting. The details of each study (recruitment, participant characteristics, and data collection methods) are described in the following sections.

## 2. Lab-Based Study Protocol

### 2.1. Data Collection

A total of 152 adult participants (48% male, age 18–64 years old) were recruited in the lab-based protocol. The recruitment method and participant eligibility criteria were similar in both lab-based and free-living protocol and accomplished via fliers, emails, and social networks (e.g., Twitter, Facebook). Interested participants completed an online screener and scheduled a lab visit to determine eligibility by performing a wide array of physical activities. Participants were screened for conditions that could limit their physical activity (e.g., cardiovascular disease, high blood pressure) as well as completed a physical activity readiness questionnaire. For both studies, informed consent was obtained from each participant prior to enrollment. The university’s institutional review board approved all study materials and procedures.

After obtaining consent, each participant was scheduled for a two-hour laboratory visit. They were instructed to wear comfortable clothing and were fitted with a GENEActiv accelerometer (Activinsights Ltd., Kimbolton, Huntingdon, UK) on their non-dominant wrist along with other activity monitors. The GENEActiv is a lightweight, waterproof, wrist-worn sensor that collects raw acceleration data. Adult participants performed a set of ambulatory and lifestyle activities randomly selected from a predetermined pool of activities (see Table 1). Participants were video-recorded completing each of the activities using a custom-designed Android app developed by our research team. This application provided automated start and stop times for each activity and an electronic video file of each activity performed. Table 1 shows the major groups of PA, the metabolic equivalent (MET) values (defined as the ratio between energy expenditure during an activity and energy expenditure at rest) associated to each PA, according to the 2011 Adult Compendium of PA [37], and their corresponding intensity levels.

### 2.2. Data Processing

**Figure 1:**
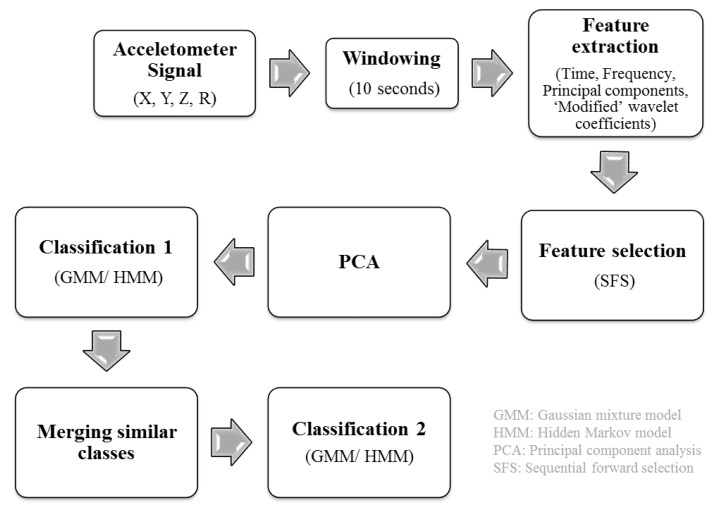
Lab-based activity classification process chain.

Tri-axial accelerometer data (*X*, *Y* and *Z* axes) were collected at a sampling rate of 100 Hz, during which the participants had to stay still for the first 5 seconds, and then perform a specific PA for a fixed period of time. As a part of pre-processing, the resultant acceleration, $R=\sqrt{X^{2}+Y^{2}+Z^{2}}$, was calculated and used as a fourth signal along with the *X*, *Y* and *Z* direction acceleration signals. After the activity transitions were identified from observed labels, the four acceleration signals were divided into windows of 10 seconds (1000 samples) without overlap, which is enough to capture both stationary and properties of the signal. To find descriptors of unique PA, various features were extracted from windowed accelerometer signals, followed by a feature selection method. Supervised classification was performed using the Gaussian mixture model (GMM) and the hidden Markov model (HMM), and their performances were compared. The entire lab-based process chain is shown in Figure 1.

#### 2.2.1. Feature Extraction

In this study, we have investigated some state-of-the-art features and introduced some novel ones as descriptors of PA for each window of accelerometer signals. 130 such features were extracted from every 10 second window (1000 samples) from different combinations of the four acceleration signals. We have briefly explained some essential backgrounds of the features investigated in this study.

- *Time-Domain Features*: Mean, standard deviation, skewness, kurtosis, energy and the squared sum of the *Y* and *Z* acceleration signals under the 25th and 5th percentile.
- *Frequency-Domain Features*: Maximum magnitude between 1→5 Hz, sum of frequency component heights below 5 Hz and number of peaks in spectrum below 5 Hz.
- *Principal Component Features*: First four principal components of *X*, *Y*, *Z* and *R*.
- *‘Modified’ Wavelet Coefficient Features*: The wavelet transform provides a time-frequency representation of a signal, as it gives an optimal resolution in both time and frequency domains [38]. In our case we used a three level Haar wavelet decomposition to extract wavelet coefficients from each 10 seconds window. We used the Kolmogorov-Smirnov (KS) test, to automatically select 20 coefficients out of 1000 (10 secs window). Given a wavelet coefficient *x*, across all the windows of a specific PA, the test compares the cumulative distribution function F(x) with that of a Gaussian distribution with the same mean and variance G(x), and hence it finds the coefficients that show maximum deviation as a sign of multi-modal distribution.

#### 2.2.2. Feature Selection

Machine learning algorithms always present problems when dealing with high dimensional inputs, so we selected the most ‘efficient’ features out of 130 features. For this purpose, we used the sequential forward selection (SFS) method. The SFS is a greedy search algorithm that works in tandem with classifiers and compares classification accuracy at each step. We used the SFS algorithm for training on a random subset of 40 adult participants’ laboratory accelerometer data, using a GMM classifier. Results showed that the ‘modified’ wavelet coefficients extracted from the resultant acceleration signal (R) were the most ‘efficient’ or highest ranked features. This suggests that the ‘modified’ wavelet coefficients of R can be used as a descriptor of unique PA. To make use of all the 20 wavelet coefficients we computed the principal components of these coefficients, and used the first 10 components as the feature space for PA classification.

#### 2.2.3. Classification

For classification of physical activities, we explored two classification algorithms, GMM and HMM, with the first 10 principal components of the ‘modified’ wavelet features, used as the input feature vector. We assume that wavelet features of each PA follows a Gaussian distribution. Based on this assumption, the principal components which are orthogonal vectors also follow Gaussian distributions. Thus, the choice of GMM and HMM with Gaussian distribution as their output distribution is justified. To measure the specificity of classification, we executed two levels of classification. The first level was used to combine similar activities and reduce the number of activity classes and the second level was used to extract the models for each activity (unique or combined).

- *Gaussian Mixture Model*: GMM is one of the most commonly used classifiers, which models the probability distribution of data as a linear combination of multiple Gaussian distributions. To create a model, the optimal values of each Gaussian distribution in the mixture must be estimated, using the Expectation-Maximization (EM) procedure [39]. GMMs have been extensively used for supervised classification problems, in which a GMM can model a single class, but can also be used for unsupervised clustering problems [40]. Before estimating the Gaussian distribution, we initialized the GMM using the Linde-Buzo-Gray (LBG) k-means algorithm [41].
- *Hidden Markov Model*: We used the GMM as the probability distribution function of the HMM output, otherwise known as the emission probability parameter. The Viterbi approximation path algorithm [42] was used to estimate the new labels for which the joint distribution of *X* (feature vector) and *Z* (observed PA labels) is maximized. The algorithm considers the most likely path instead of summing over all possible state sequences, which saves computation time.
- *Merging Similar Classes*: One shortcoming of using a single accelerometer is that there is a high possibility that similar activities (e.g., ‘*Hard surface walking, while carrying 8 lb. object*’ and ‘*Hard surface walking, while holding filled coffee cup*’) might be hard to classify. Consequently, we executed a simple method to measure the specificity of classification and find out which classes are more likely to get merged. The confusion matrix was constructed after first level of classification using the predicted and actual classes as its rows and columns, respectively. We employed a thresholding technique to combine similar classes into one larger class. For any class, if more than 50% of the class was predicted as another class, we combined them into a single class. This method helped us to find similar PAs in an unsupervised manner. After combining the similar classes, we performed a second level of classification to construct the final confusion matrix. We have shown the final number of combined classes as the measure of specificity.

### 2.3. Results

We trained the classification process with a random subset of 40 adult participants using SFS and GMM. The rest (112 adults) were used as the test data. The classification results for the adult samples are shown below.

#### 2.3.1. Lab-Based PA Classification Results

We have shown classification results for the four major classes of activities; stationary, walking, running and stair climbing in Table 2. The table shows the number of initial activity classes, the number of combined classes after merging and the final classification accuracy using GMM and HMM. Out of 15 walking activities, 5 classes (PA classes 5, 10, 12, 14 and 16 from Table 1) and two classes (PA classes 15 and 19 from Table 1) were merged into two single PA classes using GMM. With HMM, two classes (PA classes 6 and 9), four classes (PA classes 10, 12, 14 and 16) and two classes (PA class 15 and 19) were merged into three single PA classes. Similar groups of classes were merged with both classifiers. In all other activities, the classes mostly remained unmerged. Observing the PAs that were merged, we can see that most of the walking activities were merged. PA classes 5, 10, 12, 14 and 16 were walking activities with or without carrying something with their dominant hand, and 15 and 19 were both activities wearing dress shoes. Some of these merges were similar activities of different intensity. In this study, we were limited to a single accelerometer on the non-dominant wrist, which might be the reason why both the classifiers were not able to distinguish between these classes. This suggests that the features from just the non-dominant wrist accelerometer are not able to capture unique descriptors of these PAs. This limitation might be because of less variability in the intensity of movement of the non-dominant arm. However, despite such limitations, the classifiers could identify various complex activities. This suggests that the ‘modified’ wavelet features can be an accurate descriptor of physical activities. Comparing the two classifiers, the final classification accuracies for all the major classes were 99.91% (GMM) for stationary, 84.87% (HMM) for walking, 99.86% (HMM) for running and 100% (GMM) for stair climbing activities.

#### 2.3.2. Best Classification Model Selection

**Figure 2:**
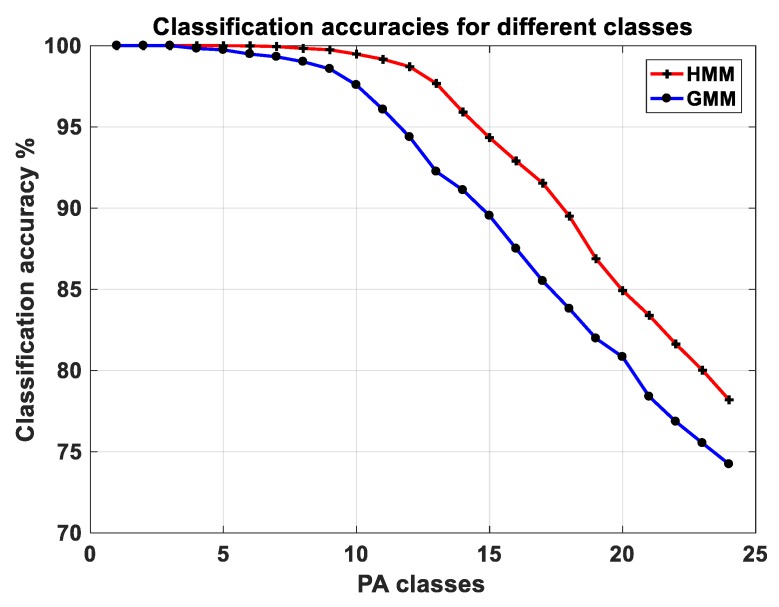
Convergence characteristics of each classifier (GMM and HMM), comparing the classification accuracies, as a PA class is merged at every step.

The best classification model was estimated by comparing the two classification algorithms. Three parameters were used to compare the performances of the classifiers: trace of the confusion matrix after the two levels of classification, final classification accuracy (mean of the trace of the final confusion matrix) and total number of classes identified after merging (specificity). Given the input feature vector of 10 principal components (of ‘modified’ wavelet features), we tested the classifiers in multiple feature spaces (2-D to 10-D, each dimension representing a principal component) to find the best classification model and feature space. The GMM gave the best classification performance in 10-D feature space with an accuracy of 78.5% (21 final classes), and HMM gave the best performance in 6-D feature space with accuracy 90.5% (17 final classes). Upon comparing both classifiers, the GMM achieved higher average trace with fewer classes combined, while the HMM achieved higher classification accuracy with more classes combined. To decide on the better classifier, we made a comparison between the two best cases of GMM and HMM by combining one class at a time and comparing the accuracy after each step, until all classes were merged to one whole class (Figure 2). It can be seen in Figure 2, as we kept combining PA classes, HMM showed a better convergence than GMM. Thus, the classification model of the HMM in 6-D feature space was selected as the best classification model. We used the models of all the 24 classes to identify PA in the free-living setting.

## 3. Free-Living Study Protocol

### 3.1. Data Collection

The 20 participants (50% male, age 21–46 years old) who participated in the free -living protocol were instructed to indicate two typical days (one weekday and one weekend day) for data collection. The participants included students, office workers, professors and home-makers. On those selected days, they were instructed to maintain their usual daily activity pattern while two researchers were independently classifying their activities through direct observation [43]. The researchers continuously classified a participant’s activity over a 6–8 hour period using a researcher-developed mobile app that allowed for continuous activity classification. Activities were labeled based on the type and context of activities. The six physical activity type labels were walking, sitting, jogging, reclining, standing and squatting whereas the context labels were sports/exercise, household chores, transportation, occupation and leisure. Approximately 8% of the data were classified as unobserved, when participants required private time (e.g., restroom use) or were out of sight of the researchers. On both days, participants were asked to wear the GENEActiv continuously along with other activity monitors.

### 3.2. Data Processing

**Figure 3:**
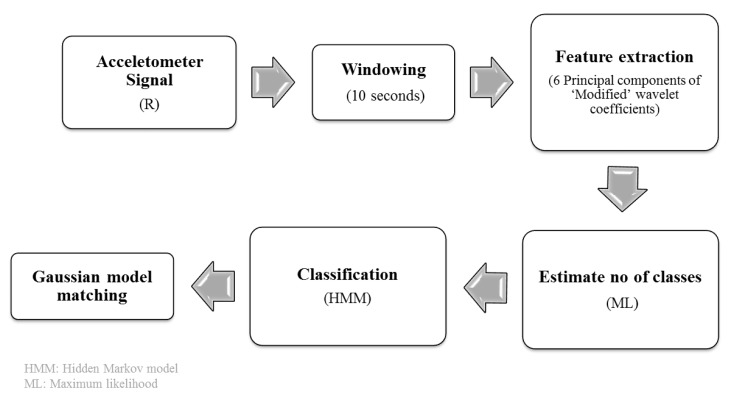
Free-living activity identification process chain.

In the lab-based settings, we modeled unique PA by distribution of Gaussian mixtures in a 6-D space. From the lab-based results, it was shown that the best supervised classification model was accomplished using HMM with the first six principal components of the ‘modified’ wavelet features. We used all the 24 PA models to identify PA in the free-living settings in an unsupervised classification framework. The primary goal of the free-living data analysis was to identify PA and the PA intensity associated to the identified activity types. The entire process was divided into the following sub-sections based on the order they were performed: pre-processing, feature extraction, unsupervised classification and Gaussian model matching (see Figure 3).

#### 3.2.1. Pre-Processing

The resultant acceleration, R, was calculated from the tri-axial acceleration signals and was used as the main signal from which features (PA descriptors) were extracted from 10 second windows of the signal.

#### 3.2.2. Feature Extraction

The ‘modified’ wavelet coefficients proved to be the most efficient feature choice for the lab-based settings study. The first six principal components of 20 ‘modified’ wavelet coefficients were used as feature vector.

#### 3.2.3. Unsupervised Classification

Since the number of activities performed during a session was unknown, we first estimated the total number of activities performed using Gaussian mixture maximum likelihood estimation. The maximum log likelihood is calculated using the following equation,

$$M_{l}=\underset{K}{arg max}\sum_{n=1}^{N}\left\{\sum_{k=1}^{K}\pi_{k}N\left(x_{n}|\mu_{k},\Sigma_{k}\right)\right\}$$

where, $x_{n}$ are the data points, *N* is total number of data points from a session and *K* is the total number of PA classes for each session. $\mu_{k}$ and $\Sigma_{k}$ are the mean and standard deviation of the Gaussian distributions corresponding to each PA class. After estimating the total number of classes, we performed classification using HMM with output distribution of Gaussian mixtures to find the Gaussian distribution corresponding to each activity.

#### 3.2.4. Gaussian Model Matching

Using unsupervised classification, we managed to estimate the total number of activities and modeled them by a mixture of Gaussians in the 6-D feature space. However, we still needed to identify the activities. We identified an unsupervised activity as the lab-based PA that had the ‘minimum distance’ in the feature space. We defined this ‘distance’ as the distance between the means of the Gaussians of the unsupervised model and the best supervised model. The predicted unsupervised PA is given by $L_{c}$ and computed as follows,

$$k_{c}=\underset{L_{c},c=1,...,24}{arg min}\left|\mu_{k}-\mu_{L_{c}}\right|$$

$\mu_{x}$ and $\mu_{L_{c}}$ are the means of the *k*th free-living PA class from the unsupervised model and cth lab-based PA class from the best supervised model Gaussian distributions.

### 3.3. Results

**Figure 4:**
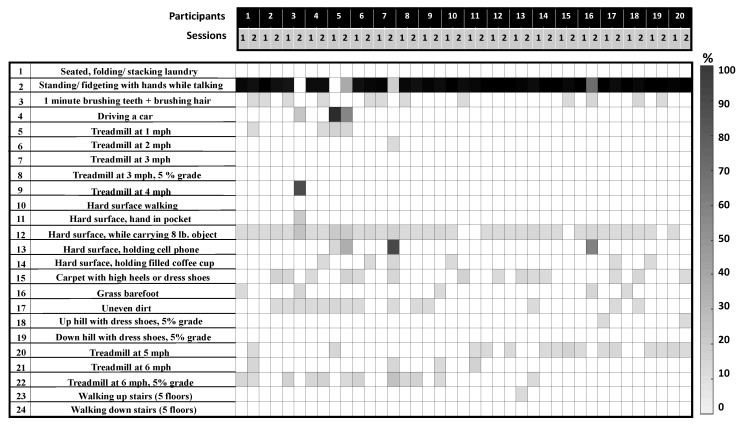
Free-living analysis results, showing the proportion of identified PA for each participant in each session; each column represents a session, showing the percentage of time spent on unique activities (out of 24 lab-based PA) by a participant in that session.

**Figure 5:**
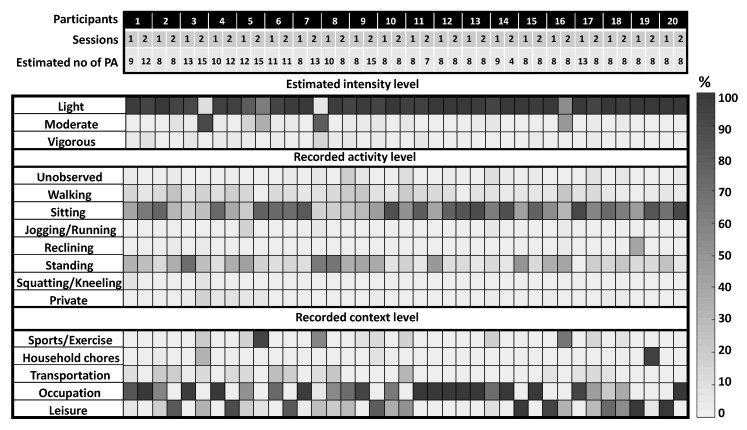
Free-living analysis results, showing the estimated number of PA and intensity levels (estimated), the observed activity types and contexts for every participant in each session. Results are shown in the form of percentage of time spent on each type of activity in a session. For example, participant 1 performed more that 90% of his 1st session performing sedentary or light intensity physical activities (estimated), and from the observed labels, it can be seen that he performed sitting and standing activities for more than 90% of the time, which was mostly during his workday.

We first estimated the total number of activities performed by a participant in each session using Gaussian mixture maximum likelihood. HMM and Gaussian model matching were used to identify the estimated activities among the pool of lab-based activities. In Figure 4, we have shown the proportion of the identified PA performed by the participants in each free-living session. It can be seen that ‘*Standing/fidgeting with hands while talking*’ was the most commonly performed PA. Most of the study participants spent the majority of their sessions performing stationary activities, and at most 10% of the time performing ambulatory activities. Result shows that participants 3, 5, 6 and 16 mostly performed ambulatory activities during their second sessions. We also estimated the intensity levels of the activities using the corresponding MET values of the estimated classes. A correlation coefficient of 0.80 was achieved between the estimated and the actual intensity level, which was approximately calculated from direct observations. Figure 5 shows the estimated number of activities performed by a participant (total 20) in each session (total 2) and the estimated intensity levels along with the recorded activity and context labels. Results are shown in terms of percentage of time spent on different activities (estimated and observed).

## 4. Discussion

This study systematically classified 24 lab-based supervised PAs and used the best classification model to identify activities in free-living settings. The lab-based participants (*N* = 152) performed activities from a pool of four stationary, 15 walking, three running and two stair climbing activities. We achieved fairly high accuracy, identifying classes from each activity group with both GMM (79–100%) and HMM (85–99%), with some limitations in specificity regarding a few walking activities. We then tested both the classifiers with the entire dataset in different feature spaces to find out the best classification space. The HMM in a 6-D feature space proved to be the best classification model and this model was used to identify unsupervised activities in the free-living settings. We estimated the total number of activities and identified them for 20 participants in each session. We further estimated the PA intensity levels with high accuracy (approximately 80%) and found that nearly all participants spent most of their time doing stationary and light intensity activities. The recorded activity levels showed that participants spent most of their time performing stationary (i.e., sitting and standing) activities.

In the last decade, GMMs and HMMs have been successfully applied in classification problems for their low computational complexity and robustness. HMMs make use of both the similarity of shapes between test and reference signals and the probabilities of shapes appearing and succeeding in time series signals, which makes it a dynamic modeling scheme. The GMM, on the other hand, is a static modeling scheme and it can be thought of as a single state HMM. In this study, the GMM does a better job classifying stationary activities, but overall the HMM outperforms GMM. This suggests that dynamic models are more suitable to recognize complex PA, especially non -stationary activities. On the other hand, a static model like GMM is more likely to classify stationary activities with better precision.

This study is unique because both lab-based and free-living dataset were investigated, with one dataset co-dependent on the other. We identified novel descriptors of PA from accelerometer signals (‘modified’ wavelet coefficients) that can be used to classify PA and produce near-accurate models. In our previous paper [34], we already showed that these features were efficient descriptors of gait patterns in 99 older adults with disabilities. With the use of a novel unsupervised classification technique we identified free-living PA and estimated energy expenditure. Generally, activities performed on a daily basis are more complex than the 24 activities investigated in this study. This study suggests that although the identified activities might not be exactly the same as the real activity, it can be a close approximation. Of note, our results suggest less degradation in activity classification accuracy from laboratory to free-living settings than previous studies. We posit that this may be because of the robust activity classifier that was developed given the large sample size and diverse set of laboratory-based activities.

This study has important implications for physical activity researchers. First, because these data were collected using a raw waveform accelerometry on the wrist, these computations can be replicated across a large range of wearable sensors that capture and make available to the researcher raw accelerations, and are not limited to the GENEactiv sensors used here. Accelerometers that can record the magnitude and intensity of movement by measuring acceleration between the magnitudes of ±8g (where *g* is equal to 9.825 ms${}^{-2}$, the acceleration of gravity), within a frequency range of 0 to 1 kHz, produce good spatiotemporal resolution. A good spatiotemporal resolution of the accelerometer waveform is sufficient to extract the ‘modified’ wavelet coefficients as descriptors of PA. Second, the study posits limitations regarding identification of some walking activities, which is due to the use of only one accelerometer on the non-dominant wrist. Although wrist-worn accelerometers are most convenient to wear and associated with greater wear-time compliance, we might be able to improve our results with the use of multiple accelerometers at various other locations of the body. Third, although ample details are provided here for data scientists to replicate this approach, the methods are not computationally intensive and more user -friendly tools are currently being prepared to make this approach available for physical activity researchers. The manufacturers of wearable sensors that are commonly used by physical activity researchers are encouraged to include this algorithm in data analysis packages they are made available to their customers.

In summary, this study contributes to the use of wearable sensors to identify physical activities and estimate energy expenditure in free-living settings by applying state-of-the-art machine learning approaches to a diverse set of laboratory -based, supervised activities. This study has demonstrated success in transferring lab-based validation techniques to the estimation of free-living activities that can be applied to future studies that wish to estimate physical activity in cohort or intervention studies.

## Figures and Tables

**Table 1:** Laboratory dataset and physical activity details, showing the unique physical activities performed by the participants, with the associated metabolic equivalent (MET) values and intensities.

Dataset (Adults)	PA No	PA Class	MET Value ${}^{a}$	Intensity Label ${}^{b}$
Stationary	1	Seated, folding/stacking laundry	2.0	L
	2	Standing/fidgeting with hands while talking	1.8	L
	3	1 minute brushing teeth + 1 minute brushing hair	2.0	L
	4	Driving a car	2.5	L
Walking	5	Treadmill at 1 mph	2.0	L
	6	Treadmill at 2 mph	2.8	L
	7	Treadmill at 3 mph	3.5	M
	8	Treadmill at 3 mph, 5% grade	5.3	M
	9	Treadmill at 4 mph	4.9	M
	10	Hard surface walking	2.8	L
	11	Hard surface, hand in pocket	3.5	M
	12	Hard surface, while carrying 8 lb. object	5.0	M
	13	Hard surface, holding cell phone	4.5	M
	14	Hard surface, holding filled coffee cup	3.5	M
	15	Carpet with high heels or dress shoes	2.8	L
	16	Grass barefoot	4.8	M
	17	Uneven dirt	4.5	M
	18	Uphill with high heels or dress shoes, 5% grade	5.3	M
	19	Downhill with high heels or dress shoes, 5% grade	3.3	M
Running	20	Treadmill at 5 mph	8.3	V
	21	Treadmill at 6 mph	9.8	V
	22	Treadmill at 6 mph, 5% grade	12.3	V
Stair climbing	23	Walking upstairs (5 floors)	4.0	M
	24	Walking down stairs (5 floors)	3.5	M

${}^{a}$ MET values obtained from the Adults Compendium of PA, Ainsworth et al. 2011; ${}^{b}$ L = light intensity, M = moderate intensity, V = vigorous intensity.

**Table 2:** Classification accuracy for various lab-based physical activities using Gaussian mixture model (GMM) and hidden Markov model (HMM) (showing the initial and the final number of PA classes after merging).

Activities	Original PA Classes	PA Classes after Merging (GMM)	PA Classes after Merging (HMM)	Classification Accuracy% (GMM)	Classification Accuracy% (HMM)
Stationary	4	4	4	99.91	89.32
Walking	15	10	10	79.57	84.87
Running	3	2	3	91.4	99.86
Stair-climbing	2	2	2	100	99.8
All activities	24	21	17	78.54	90.2

## Supplementary Materials

The following are available online at http://www.mdpi.com/1424-8220/18/11/3893/s1, Section 1: Summary of features extracted for lab-based activity classification, Section 2: Sequential Forward Selection Algorithm, Section 3: Gaussian mixture model, Section 4: Hidden Markov model, ection 5: Class merging using confusion matrix, Section 6: Lab-based classifier comparisons.

## References

[1] Choo J., Elci O.U., Yang K., Turk M.W., Styn M.A., Sereika S.M., Music E., Burke L.E. (2010). Longitudinal relationship between physical activity and cardiometabolic factors in overweight and obese adults. Eur. J. Appl. Physiol..

[2] Craig S.B., Bandini L.G., Lichtenstein A.H., Schaefer E.J., Dietz W.H. (1996). The impact of physical activity on lipids, lipoproteins, and blood pressure in preadolescent girls. Pediatrics.

[3] Ekelund U., Franks P.W., Sharp S., Brage S., Wareham N.J. (2007). Increase in physical activity energy expenditure is associated with reduced metabolic risk independent of change in fatness and fitness. Diabetes Care.

[4] U.S. Department of Health and Human Services (2008). 2008 Physical Activity Guidelines for Americans.

[5] Bobick A.F., Davis J.W. (2001). The recognition of human movement using temporal templates. IEEE Trans. Pattern Anal. Mach. Intell..

[6] Veeraraghavan A., Roy-Chowdhury A.K., Chellappa R. (2005). Matching shape sequences in video with applications in human movement analysis. IEEE Trans. Pattern Anal. Mach. Intell..

[7] Duong T.V., Bui H.H., Phung D.Q., Venkatesh S. Activity recognition and abnormality detection with the switching hidden semi-Markov model. Proceedings of the 2005 IEEE Computer Society Conference on Computer Vision and Pattern Recognition (CVPR 2005).

[8] Liu L., Wang S., Su G., Huang Z.G., Liu M. (2017). Towards complex activity recognition using a Bayesian network-based probabilistic generative framework. Pattern Recognit..

[9] Liu L., Wang S., Hu B., Qiong Q., Wen J., Rosenblum D.S. (2018). Learning structures of interval-based Bayesian networks in probabilistic generative model for human complex activity recognition. Pattern Recognit..

[10] Schüldt C., Laptev I., Caputo B. Recognizing human actions: A local SVM approach. Proceedings of the 17th International Conference on Pattern Recognition.

[11] Mannini A., Rosenberger M., Haskell W.L., Sabatini A.M., Intille S.S. (2017). Activity recognition in youth using single accelerometer placed at wrist or ankle. Med. Sci. Sports Exerc..

[12] Chowdhury A.K., Tjondronegoro D., Chandran V., Trost S.G. (2017). Ensemble Methods for Classification of Physical Activities from Wrist Accelerometry. Med. Sci. Sports Exerc..

[13] Dinger M.K., Behrens T.K. (2006). Accelerometer-determined physical activity of free-living college students. Med. Sci. Sports Exerc..

[14] De Vries S.I., Engels M., Garre F.G. (2011). Identification of children’s activity type with accelerometer-based neural networks. Med. Sci. Sports Exerc..

[15] Hikihara Y., Tanaka C., Oshima Y., Ohkawara K., Ishikawa-Takata K., Tanaka S. (2014). Prediction models discriminating between nonlocomotive and locomotive activities in children using a triaxial accelerometer with a gravity-removal physical activity classification algorithm. PLoS ONE.

[16] Del Rosario M.B., Wang K., Wang J., Liu Y., Brodie M., Delbaere K., Lovell N.H., Lord S.R., Redmond S.J. (2014). A comparison of activity classification in younger and older cohorts using a smartphone. Physiol. Meas..

[17] Hansen B.H., Kolle E., Dyrstad S.M., Holme I., Anderssen S.A. (2012). Accelerometer-Determined Physical Activity in Adults and Older People. Med. Sci. Sports Exerc..

[18] Welch W.A., Bassett D.R., Thompson D.L., Freedson P.S., Staudenmayer J.W., John D., Steeves J.A., Conger S.A., Ceaser T., Howe C.A. (2013). Classification accuracy of the wrist-worn gravity estimator of normal everyday activity accelerometer. Med. Sci. Sports Exerc..

[19] Dong B., Montoye A.H.K., Pfeiffer K.A., Biswas S. (2013). Energy-aware activity classification using wearable sensor networks. Sensing Technologies for Global Health, Military Medicine, and Environmental Monitoring.

[20] Mannini A., Intille S.S., Rosenberger M., Sabatini A.M. (2014). Activity Recognition Using a Single Accelerometer Placed at the Wrist or Ankle. Med. Sci. Sports Exerc..

[21] Skotte J., Korshøj M., Kristiansen J., Hanisch C., Holtermann A. (2014). Detection of Physical Activity Types Using Triaxial Accelerometers. J. Phys. Act. Health.

[22] Zhang S., Rowlands A.V., Murray P., Hurst T.L. (2012). Physical activity classification using the GENEA wrist-worn accelerometer. Med. Sci. Sports Exerc..

[23] Rosenberg D., Godbole S., Ellis K., Di C., Lacroix A., Natarajan L., Kerr J. (2017). Classifiers for Accelerometer-Measured Behaviors in Older Women. Med. Sci. Sports Exerc..

[24] Sasaki J.E., Hickey A.M., Staudenmayer J.W., John D., Kent J.A., Freedson P.S. (2016). Performance of activity classification algorithms in free-living older adults. Med. Sci. Sports Exerc..

[25] Tedesco S., Barton J., O’Flynn B. (2017). A review of activity trackers for senior citizens: Research perspectives, commercial landscape and the role of the insurance industry. Sensors.

[26] Freedson P.S., Melanson E., Sirard J. (1998). Calibration of the Computer Science and Applications, Inc. accelerometer. Med. Sci. Sports Exerc..

[27] Montoye A.H., Mudd L.M., Biswas S., Pfeiffer K.A. (2015). Energy Expenditure Prediction Using Raw Accelerometer Data in Simulated Free Living. Med. Sci. Sports Exerc..

[28] Wang Q., Lohit S., Toledo M.J., Buman M.P., Turaga P. A statistical estimation framework for energy expenditure of physical activities from a wrist-worn accelerometer. Proceedings of the Annual International Conference of the IEEE Engineering in Medicine and Biology Society (EMBS).

[29] Sirichana W., Dolezal B.A., Neufeld E.V., Wang X., Cooper C.B. (2017). Wrist-worn triaxial accelerometry predicts the energy expenditure of non-vigorous daily physical activities. J. Sci. Med. Sport.

[30] Tapia E.M., Intille S.S., Haskell W., Larson K.W.J., King A., Friedman R. Real-Time Recognition of Physical Activities and their Intensitiies Using Wireless Accelerometers and a Heart Monitor. Proceedings of the International Symposium on Wearable Computers.

[31] Ellis K., Kerr J., Godbole S., Staudenmayer J., Lanckriet G. (2016). Hip and wrist accelerometer algorithms for free-living behavior classification. Med. Sci. Sports Exerc..

[32] Trost S.G., Wong W.K., Pfeiffer K.A., Zheng Y. (2012). Artificial Neural Networks to Predict Activity Type and Energy Expenditure in Youth. Med. Sci. Sports Exerc..

[33] Dutta A., Ma O., Buman M.P., Bliss D.W. Learning approach for classification of GENEActiv accelerometer data for unique activity identification. Proceedings of the 13th Annual Body Sensor Networks Conference (BSN 2016).

[34] Dutta A., Ma O., Toledo M., Buman M.P., Bliss D.W. Comparing Gaussian mixture model and hidden Markov model to classify unique physical activities from accelerometer sensor data. Proceedings of the 2016 15th IEEE International Conference on Machine Learning and Applications (ICMLA 2016).

[35] Semwal V.B., Singha J., Sharma P.K., Chauhan A., Behera B. (2017). An optimized feature selection technique based on incremental feature analysis for bio-metric gait data classification. Multimed. Tools Appl..

[36] Pavey T.G., Gilson N.D., Gomersall S.R., Clark B., Trost S.G. (2017). Field evaluation of a random forest activity classifier for wrist-worn accelerometer data. J. Sci. Med. Sport.

[37] Ainsworth B.E., Haskell W.L., Herrmann S.D., Meckes N., Bassett D.R., Tudor-Locke C., Greer J.L., Vezina J., Whitt-Glover M.C., Leon A.S. (2011). 2011 compendium of physical activities: A second update of codes and MET values. Med. Sci. Sports Exerc..

[38] Barford L.A., Fazzio R.S., Smith D.R. (1992). An Introduction to Wavelets.

[39] Moon T. (1996). The expectation-maximization algorithm. IEEE Signal Process. Mag..

[40] Reynolds D.A., Rose R.C. (1995). Robust Text-Independent Speaker Identification Using Gaussian Mixture Speaker Models. IEEE Trans. Speech Audio Process..

[41] Ortega J.P., del Rocio Boone Rojas M., Somodevilla Garcia M.J. Research issues on K-means Algorithm: An Experimental Trial Using Matlab. Proceedings of the 2nd Workshop on Semantic Web and New Technologies.

[42] Forney G.D. (1973). The viterbi algorithm. Proc. IEEE.

[43] Florez Pregonero A.A. (2017). Monitors-Based Measurement of Sedentary Behaviors and Light Physical Activity in Adults. Ph.D. Thesis.

