# Supplementary material for: Identifying Free-Living Physical Activities Using Lab-Based Models with Wearable Accelerometers
Source: Sensors (Basel). 2018 Nov 12;18(11):3893. doi: 10.3390/s18113893 (PMC6263387; doi:10.3390/s18113893)
Supplement: Supplementary file 1 [file sensors-18-03893-s001.pdf]

Article

Supplementary Material for "Identifying Free-Living Physical Activities Using Lab-Based Models With Wearable Accelerometers"

Arindam Dutta [1], Owen Ma [1], Meynard Toledo [2], Alberto Florez Pregonero [3], Barbara E. Ainsworth [2], Matthew P. Buman [2] and Daniel W. Bliss [1]

[1] School of Electrical, Computer and Energy Engineering, Arizona State University, Tempe AZ; adutta7@asu.edu
[2] College of Health Solutions, Arizona State University, Phoenix AZ
[3] Departamento de Formación, Pontificia Universidad Javeriana,Bogotá D.C., 110231, Colombia;

Version November 11, 2018 submitted to Journal Not Specified

1. Summary of Features Extracted for Lab-Based Activity Classification

An accelerometer signal consists of three components; X, Y and Z. These signals have been used to classify physical activity, estimate energy expenditure, study gait patterns etc. Various time and frequency domain features are extracted from each components of acceleration. Features have also been extracted from the resultant acceleration of

- X, Y and Z:

$$R = \sqrt{X^2 + Y^2 + Z^2}$$

  and
- Y and Z:

$$T = \sqrt{Y^2 + Z^2}$$

We have listed the features (Table 1) that were explored in this paper.

- **Time-domain features**

  Time-domain features like mean, standard deviation, percentiles were previously used as important activity features. We have used some the same features and did a few extensions. We have employed seven sets of time-domain features namely mean, standard deviation, skewness, kurtosis, energy, squared sum of data under 25 percentile, squared sum of data under 75 percentile. The squared sum of data under 25 and 75 percentiles are extracted only from T. So, 22 different time domain features were used to represent each window.

- **Frequency-domain features**

  For frequency-domain feature fast Fourier transform (FFT) was applied on each window and three sets of features were extracted. It has been observed that most of the signal strength lies between 0-15 Hz, with a bulk of peaks below 5 Hz. Keeping that in mind features like the frequency with maximum magnitude between 1-5 Hz, sum of height of frequency component below 5 Hz, and number of peaks in spectrum below 5 Hz.

Table 1. Summary of features extracted

No. of features	Feature domain	Description	Signals used
4	Time	Mean	X, Y, Z, R
4	Time	Standard deviation	X, Y, Z, R
4	Time	Skewness	X, Y, Z, R
4	Time	Kurtosis	X, Y, Z, R
4	Time	Energy	X, Y, Z, R
1	Time	Squared sum of data under 25 percentile	T
1	Time	Squared sum of data under 75 percentile	T
4	Frequency	Maximum frequency	X, Y, Z, R
4	Frequency	Sum of heights of frequency components below 5Hz	X, Y, Z, R
4	Frequency	Number of peaks in spectrum below 5Hz	X, Y, Z, R
16	Principal components	PCA (1,2,3,4)	X, Y, Z, R
80	time-frequency	'Modified' Wavelet coefficients	X, Y, Z, R

- **Principal component features**

  Principal component analysis (PCA) is a method widely used for dimension reduction. It takes the matrix of n windows with $p = 1000$ samples which are correlated and summarizes it by uncorrelated components also known as principal components that are linear combinations of the original p variables. The first k components display as much as possible of the variation among objects. The new principal components have a variance equal to their corresponding eigen values with the first principal component having the maximum eigen value. We have used four principal components per window for the acceleration in each axis and the resultant.
- **Modified wavelet features**

  Wavelet transform provides a time-frequency representation of the signal as it gives an optimal resolution in both time and frequency domains [1]. It is defined as convolution between the signal and a wavelet function. With this approach, the original time-domain signal (maximum frequency f) is initially decomposed into a coarse approximation and detail information by low-pass filtering (bandpass $[0, f/2]$) and high-pass filtering (bandpass $[f/2, f]$), respectively. With wavelet decomposition, the half-band filters are designed to enable perfect reconstruction of the original signal and to avoid aliasing effects. In subsequent levels of decomposition, the approximation signal from the previous level is split into a second approximation and a detail coefficient. This process is repeated to the desired decomposition level.In our case we have used a 3 level Haar wavelet decomposition, which has one of the simplest functions shown below:

$$\psi(t) = \begin{cases} 1, & \text{if } 0 \leq t < \frac{1}{2} \\ -1, & \text{if } \frac{1}{2} \leq t < 1 \\ 0 & \text{otherwise} \end{cases}$$

  After decomposition we got 1000 coefficients for each window. Since all the coefficients are not useful features, 20 coefficients were selected using the Kolmogorov Smirnov (KS) test. The KS test quantifies a distance between the empirical distribution function of the sample and the cumulative distribution function of the reference distribution, or between the empirical distribution functions of two samples [2]. Here we have used the criteria which quantifies

the deviation of a coefficient from normality. Given a coefficient x across all the windows, the test compares the cumulative distribution function of the data $F(x)$ with that of a Gaussian distribution with the same mean and variance $G(x)$. The deviation from normality is quantified by

$$max(|F(x) - G(x)|)$$

2. Sequential Forward Selection Algorithm

Sequential Forward Selection (SFS) is a popular and simple algorithm for feature selection. It is a greedy search algorithm, ideal for cases where it is desirable to minimize the number of features. The algorithm starts with an empty set of features and iteratively adds new features to the optimal set, in order to maximize a certain objective function. It works in conjunction with the classification or clustering method, updating the optimal subset of features after testing each model generated by a given subset of features. A common objective function is given by:

$$\mathrm{rr}(\boldsymbol{P}) = \frac{\sum_{c=1}^{C} \mathrm{rr}_c(\boldsymbol{P})}{C},$$

where rr_c is the recognition rate of the cluster c. In other words, the objective function is given by the mean of the main diagonal of the confusion matrix. A classifier is required to compute the confusion matrix at every point. Figure 1 shows the block diagram for SFS.

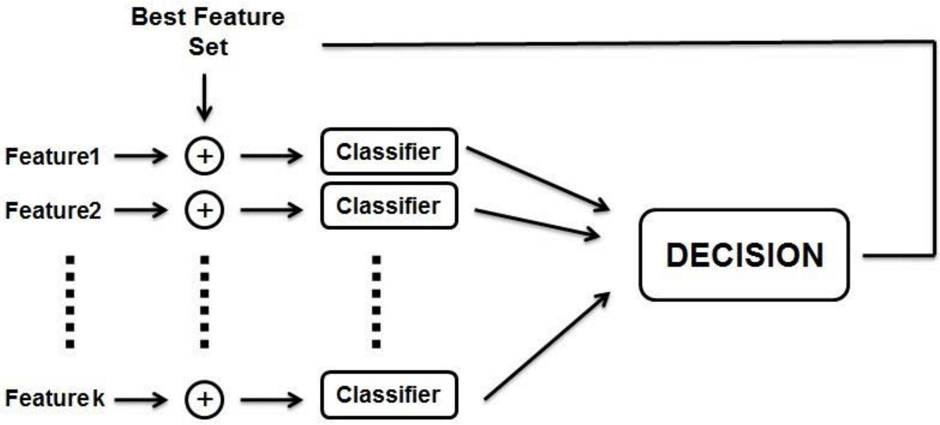

Figure 1. Block Diagram for SFS

3. Gaussian Mixture Model

Gaussian mixture models (GMM) are one of the most commonly used classifier, which are statistical models where the distribution of data takes the form of a mixture, i.e, a linear combination of several probability density functions. In a GMM, the basic probability functions are multivariate Gaussians, where the probability of the mixture is given by:

$$P(x) = \sum_{m=1}^{k} a_m \mathcal{N}(\mu_m, \Sigma_m),$$

where M is the number of Gaussians in the mixture and a_m, μ_m, Σ_m denote the weight, mean and covariance matrix for each gaussian m.

GMMs have been extensively used for supervised classification problems, in which a single GMM can model a class. To create a model, one needs to find the optimal values for a, μ and Σ for all Gaussians in the mixture, and this task is accomplished here by using the Expectation-Maximization (EM) procedure [3].

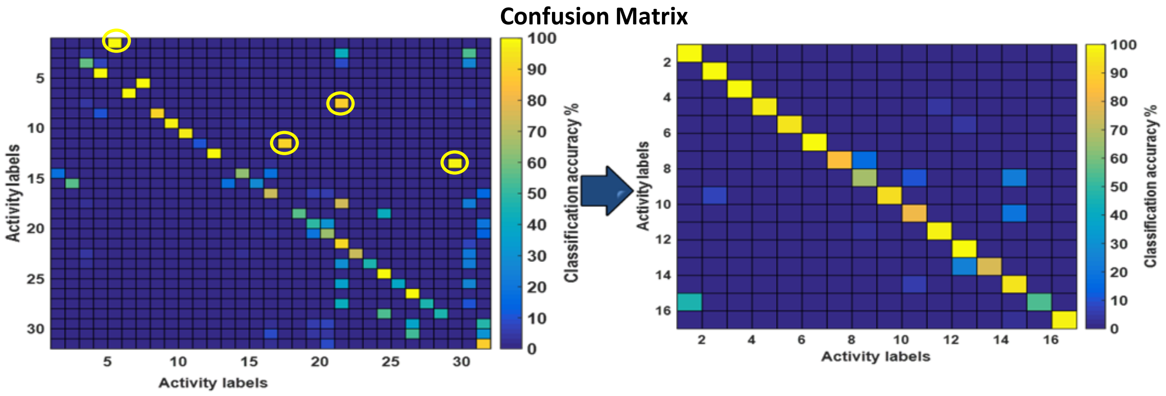

Figure 2. An example showing how similar classes were merged. The left confusion matrix is obtained after first level of classification, the confused classes are circled and are merged. The final confusion matrix after second level classification is shown on the right hand side.

One shortcoming of this method is assuming that each class has a Gaussian distribution, which is seldom the case. Besides, this method is very sensitive to a good initialization for the Gaussian parameters which is usually performed by first applying the K-means algorithm. K-means is one the simplest unsupervised learning algorithms and it is a fast, robust, easier to understand and relatively efficient algorithm that gives best result when data is distinct or well separated. However, it has some disadvantages like its inability to handle noisy data, outliers and non-linear data. Also there's always the need to specify the number of clusters apriori and initialize them properly. These centers should be placed in a cunning way because different locations generate different results. This means random intialization cannot be used if the goal is to compare different approaches, like what is done on the SFS algorithm. One way to overcome random initialization is to use the Linde-Buzo-Gray (LBG) algorithm [4], which is a slight modification of the generic K-means algorithm, in which initially just one center (the sample mean) is selected, and centers are iteratively divided until the desired number of centroids is achieved. For this reason, this algorithm only works with M center values which are a power of 2. In this study , we used a modified version of the LBG algorithm, where the center division is performed differently for each number of gaussians M. For M values that are a power of 2, the center division is given by:

$$\mu_1 = \mu_0 + \epsilon.\sigma_0$$
$$\mu_2 = \mu_0 - \epsilon.\sigma_0,$$

where μ_0 is the split cluster's center and σ_0 is the cluster's standard deviation. ϵ was empirically set to 0.4 in this study. For M values that are a power of 3, the expression is modified to:

$$\mu_1 = \mu_0$$
$$\mu_2 = \mu_0 + \epsilon.\sigma_0$$
$$\mu_3 = \mu_0 - \epsilon.\sigma_0.$$

For other M values, only the center with the highest standard deviation is chosen to be split, and this operation is repeated until the desired number M is achieved.

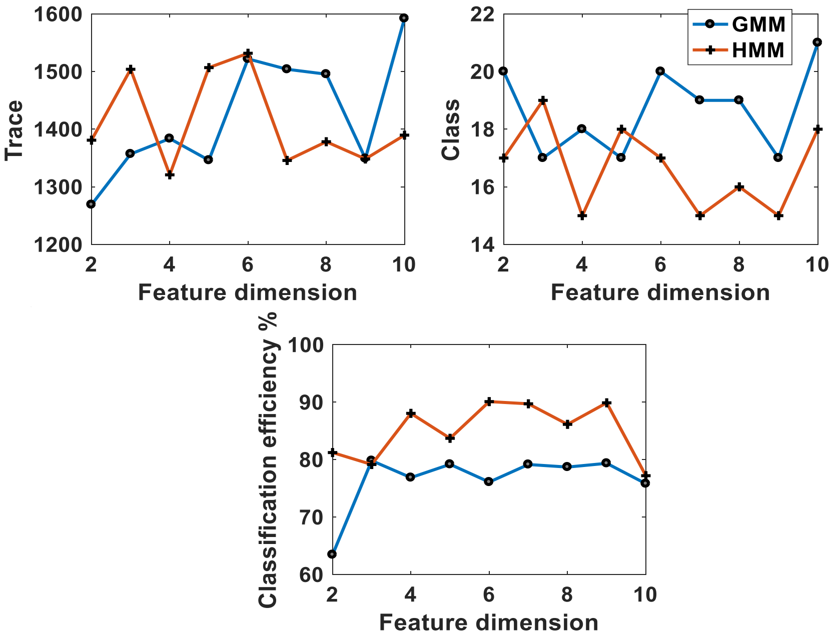

Figure 3. Comparison between GMM and HMM performance

4. Hidden Markov Model

Hidden Markov Models (HMM) are statistical models related to Markov processes. In this model, the occurrence of a sequence of events is due to the events having gone through a sequence of (hidden) states, each emitting an observation. This way, a HMM is defined by a model $\lambda = [Q, A, \pi, b_q(x)]$, where Q is the number of states, A is the state transition matrix, π is the initial state probability vector and $b_q(x)$ is the observation pdf for each state q. The optimal values for these parameters are estimated from the data using the Baum-Welch algorithm [5], which is a modification of the Expectation maximization (EM) algorithm. Like GMM, HMM can be used for supervised and unsupervised classification problems, where the former models each class as a single λ and, the latter, as a state. HMM gives the ability to model each class as Gaussian mixtures (GMs) for unsupervised case.

For input $x_1, ..., x_n$, the labels corresponding to x_i, $\hat{z_1}, ..., \hat{z_n}$ are estimated for which the joint distribution of x_i and z_i is maximum, given by,

$$\{\hat{z_1}, ..., \hat{z_n}\} = \underset{\{z_1,...,z_n\}}{\arg\max}\, p(x_1, ..., x_n, z_1, ..., z_n)$$

where joint probability is given by,

$$p(x_1, ..., x_n, z_1, ..., z_n) = \prod_1^n q(z_i|z_{i-2}, z_{i-1}) \prod_1^n e(x_i|z_i)$$

Here, we have used the GMs as the probability distribution function of the HMM output. So the output distribution is a single multivariate Gaussian with mean μ_j and covariance matrix Σ_j.

$$e(x|z_i) = N(x; \mu_j, \Sigma_j)$$

As discussed in the previous section, the Expectation Maximization algorithm is used to estimate the parameters of the above distribution. We have used the Viterbi approximation process [6] to calculate,

$$\arg\max_{\{z_1,...,z_n\}} p(x_1, ..., x_n, z_1, ..., z_n),$$

which just considers the most likely path, instead of summing over all possible state sequences.

5. Class Merging using Confusion Matrix

We used class merging to assess the specificity of the classification algorithms. Figure 2 shows a cartoon which exhibits the class merging process. From the initial confusion matrix (after first level classification), we find out the PA classes that are confused ($\geq$ 50%), and we combine them and perform classification again to find the final confusion matrix.

6. Lab-Based Classifier Comparison

Three parameters were used to compare the performances of the classifiers in the lab-based study; trace of the confusion matrix after the two levels of classification, final classification accuracy (mean of the trace of the final confusion matrix) and total number of classes identified after merging as shown in Figure 3.

References

1. Barford, L.A.; Fazzio, R.S.; Smith, D.R. An introduction to wavelets. *Hewlett-Packard Labs, Bristol, UK, Tech. Rep. HPL-92-124* **1992**, *2*, 1–29. doi:10.1109/99.388960.
2. Smirnov, N. Table for Estimating the Goodness of Fit of Empirical Distributions. *The Annals of Mathematical Statistics* **1948**. doi:10.1214/aoms/1177730256.
3. Moon, T. The expectation-maximization algorithm. *IEEE Signal Processing Magazine* **1996**, *13*, 47–60. doi:10.1109/79.543975.
4. Ortega, J.P.; del Rocio Boone Rojas, M.; Somodevilla Garcia, M.J. Research issues on K-means Algorithm : An Experimental Trial Using Matlab. *Proceedings of the 2nd Workshop on Semantic Web and New Technologies* **2009**, pp. 83–96.
5. Rabiner, L.; Juang, B. An introduction to hidden Markov models. *IEEE ASSP Magazine* **1986**. doi:10.1109/MASSP.1986.1165342.
6. Forney, G.D., J. The viterbi algorithm. *Proceedings of the IEEE* **1973**, *61*, 302–309. doi:10.1109/PROC.1973.9030.

© 2018 by the authors. Submitted to *Journal Not Specified* for possible open access publication under the terms and conditions of the Creative Commons Attribution (CC BY) license (http://creativecommons.org/licenses/by/4.0/).
